# Editorial: Improving children's oral health

**DOI:** 10.3389/froh.2025.1636820

**Published:** 2025-06-18

**Authors:** Phoebe P. Y. Lam, Duangporn Duangthip

**Affiliations:** ^1^ Faculty of Dentistry, The University of Hong Kong, Hong Kong, Hong Kong SAR, China; ^2^ College of Dentistry, The Ohio State University, Columbus, OH, United States

**Keywords:** early childhood caries (ECC), children, oral health, dentistry, dental caries, prevention

**Editorial on the Research Topic**
Improving children's oral health

Improving oral health among children worldwide has been a significant challenge. Despite efforts over the past decades, the prevalence of dental caries has shown limited improvement (1). Approximately half of children under the age of 6 are affected by dental caries (1), and untreated dental caries continues to be one of the most prevalent diseases in the adult population (2). Dental caries can have detrimental and severe impacts on children. In addition to causing pain and infection, the daily activities and quality of life of children can be significantly compromised (3). The effects can be prolonged and have a lasting impact on their permanent dentition (4). Children who present with dental caries in their primary dentition are more likely to develop dental caries in their permanent dentition (4). The impact can also extend to their general health, as children who suffer from oral diseases are more likely to experience compromised growth and development (3).

There is also a concerning issue of oral health disparity, with dental caries and other diseases being disproportionately concentrated among children from lower socioeconomic backgrounds (5). The increasing costs of dental treatments further exacerbate the situation (6), as those who are most susceptible to oral diseases are less likely to afford the necessary treatment. To enhance oral health among children and improve the condition, collaborative efforts from all stakeholders are necessary to advocate for improved oral health outcomes among children and adolescents. The five research articles focusing on the theme *“Improving Children's Oral Health”* offer various perspectives on the recent advancements in enhancing oral health among children, as well as identifying existing knowledge gaps. These articles provided valuable suggestions for appropriate directions to meet current objectives in improving children's oral health.

Masaebi et al. adopted a public health macro-perspective in assessing disease risk factors and identifying children at high risk of developing dental caries. This approach allows for focused preventive measures to be implemented to reduce the need for more complex treatments. Masaebi et al. utilized the Random Forest Algorithm to identify early signs of permanent dental caries, emphasizing factors such as maternal education and frequent consumption of sweets as indicators of increased caries risk. These findings guide policymakers and dental professionals to intervene proactively and develop targeted policies for early intervention.

Wang et al. then examined the perspective of care providers and assessed the current knowledge and skills of dental professionals regarding the placement of stainless steel crowns using the Hall technique. Through appropriate case selection, the placement of stainless steel crowns using the Hall technique has been shown to enhance children's oral health-related quality of life by reducing pain and anxiety, shortening operative time, and improving patient comfort in the long term (7). However, the potential benefits of this approach may not be fully realized if it is not widely adopted by dental practitioners. According to Wang et al., the Hall technique was not commonly practiced among pediatric dentists in China, with the main barriers being insufficient clinical knowledge on case selection and application.

According to the findings of Wang et al., the Hall technique is not adequately emphasized in undergraduate teaching, with most individuals acquiring this knowledge through postgraduate or vocational training programs. This highlights the importance of involving educators and academics in addressing the challenges of oral diseases among children. Educators play a crucial role by providing effective continuous professional development and training on modern preventive and operative approaches to healthcare professionals at the forefront.

In addition to staying current on the latest management techniques for oral diseases, dental professionals should also be knowledgeable about handling rare conditions. The case report by Varriano et al. discusses the rare occurrence of bilateral molar natal teeth, which, although uncommon, can impact an infant's nutrition and diet. Effectively managing these conditions can lead to significant improvements in oral health outcomes and provide reassurance and support to their families. Furthermore, it emphasizes the importance of empowering parents and caregivers in the fight against oral diseases as part of the broader effort to enhance children's oral health.

The interconnectedness of oral health and overall health should not be overlooked. It is crucial to leverage the expertise and collaboration of medical professionals in identifying pediatric patients with medical conditions that are closely associated with dental caries and other diseases. The rising prevalence of obesity among children and adolescents is a significant concern. Lopez Del Valle et al. sought to clarify the relationship between caries and obesity, providing valuable insights into the potential link between oral health and metabolic diseases. This research enhances our understanding and underscores the importance of collaborating with medical professionals to enhance the oral and overall health of children.

Last but not least, pediatric patients themselves are crucial stakeholders and active participants in efforts to enhance oral health among them. It is essential to ensure that children and adolescents are well-informed about methods to improve their oral health. Kassim and Alsharif investigated factors related to the dental knowledge of tooth brushing with fluoridated toothpaste among high school students. This accentuated the knowledge gaps among adolescents and shed light on ways to bolster our battle against oral diseases by empowering them to actively participate in improving their oral health.

In conclusion, this research topic emphasizes the importance of collaboration among various stakeholders in addressing the global challenges of dental caries and other oral diseases. Healthcare professionals, educators, researchers, and policymakers should unite in a concerted effort to enhance child oral health on a global scale. The articles in this research topic provide valuable insights and highlight gaps that require collective action from all stakeholders to improve dental care for future generations.
